# Systemic Lupus Erythematosus-Associated Serositis Managed With Intravenous Belimumab: A Case Report

**DOI:** 10.7759/cureus.22639

**Published:** 2022-02-26

**Authors:** Srikanth Mukkera, Maneesh Mannem, Karthik Chamarti, Leela Pillarisetty, Sai Swarupa Vulasala, Lakshmi Alahari, Anusha Ammu, Akshathh Mukkera, Rajeev K Vadlapatla

**Affiliations:** 1 Internal Medicine, Texas Tech University Health Sciences Center, Odessa, USA; 2 Obstetrics and Gynecology, Texas Tech University Health Sciences Center, Odessa, USA; 3 Radiology, University of Florida College of Medicine, Jacksonville, USA; 4 Medicine, Odessa High School, Odessa, USA; 5 Medicine, Trinity School, Midland, USA

**Keywords:** prednisone, mycophenolate, sle, benlysta, belimumab, systemic lupus erythematosus, serositis

## Abstract

Systemic lupus erythematosus (SLE) is an autoimmune disease that involves multiple organ systems. Due to the heterogeneity of its presentation, it is challenging for clinicians to diagnose and manage the symptoms. SLE has a wide range of presentations from mild to severe and involves various organ systems like mucocutaneous, musculoskeletal, cardiopulmonary, renal, gastrointestinal, and central nervous system.

Various novel treatment modalities are being used based on clinical presentation. Prednisone and methylprednisolone are commonly used as needed for acute flares of SLE. Some patients may need a low dose of oral prednisone to keep their SLE under control, which carries a risk of coronary artery disease (CAD) and many other metabolic side effects of steroids. Other long-term medications that are commonly used include hydroxychloroquine, methotrexate, azathioprine, mycophenolate, cyclosporine, and cyclophosphamide. Intravenous cyclophosphamide is used only in severe lupus with renal, pulmonary, or CNS involvement.

Rituximab is a human monoclonal B-cell cluster of differentiation (CD)20 receptor antibody used for severe SLE not responding with other medications. Other newer medications are belimumab and anifrolumab. Anifrolumab is a fully human monoclonal antibody that binds to subunit 1 of the type I interferon receptor.

We present a case of a 25-year-old female with a chronic history of SLE presented to the outpatient clinic with abdominal distension that needed frequent abdominal paracenteses. She was using hydroxychloroquine, mycophenolate mofetil, and prednisone, but her symptoms were not adequately controlled. After we started the patient on monthly intravenous belimumab, her symptoms and the frequency of visits for paracentesis gradually reduced. B-cells are known to play an essential role in the pathogenesis of SLE, and the use of belimumab, an anti-BLys (B-lymphocyte stimulator) human monoclonal antibody that inhibits B-cell growth, can play a significant role in the management of SLE associated chronic serositis.

## Introduction

Systemic lupus erythematosus (SLE) is an autoimmune disease of unknown etiology with a plethora of clinical manifestations and immunological abnormalities. SLE is predominantly seen in females with a female to male ratio of around 9:1 [1,2]. The clinical manifestations can range from constitutional symptoms such as fever, fatigue, and weight loss to the involvement of cutaneous, musculoskeletal, renal, respiratory, cardiovascular systems, with hematological, and neuropsychiatric manifestations [3-5].

The management of a patient with SLE depends on the presentation, severity, and response to medications. Medications include hydroxychloroquine, nonsteroidal anti-inflammatory drugs (NSAIDs), corticosteroids, azathioprine, methotrexate, cyclophosphamide, cyclosporine, and monoclonal antibodies such as rituximab, anifrolumab, and belimumab are used for the treatment [6]. Belimumab has been approved for the management of autoantibody-positive SLE patients with active disease, and it has been demonstrated to be clinically effective [7,8]. Belimumab is effective as adjunctive therapy in SLE patients with mucocutaneous and musculoskeletal symptoms. Its role in treating a case of chronic serositis due to SLE has not been reported before. We would like to report a case as such.

## Case presentation

A 25-year-old African American female with a past medical history of SLE with lupus nephritis and anti-phospholipid antibody syndrome presented to the outpatient clinic with abdominal distention requiring frequent paracentesis. She also has a history of malar rash, diffuse thinning of hair, photosensitive rash on the upper extremities, joint pain, and swelling. She was on hydroxychloroquine 200 mg p.o. (peroral) two times daily, mycophenolate mofetil 1.5 gm p.o. two times daily, and prednisone 10 mg p.o. daily at the time of presentation. On physical examination, her vitals were normal. Skin examination revealed diffuse thinning of hair, healed malar rash, and a discoid rash. Lungs were clear with normal breath sounds on auscultation. Heart sounds were normal without any murmurs, rubs, or gallops. The abdomen was soft and moderately distended with shifting dullness, and normal bowel sounds. The musculoskeletal examination was normal without any joint swelling or tenderness.

**Figure 1:**
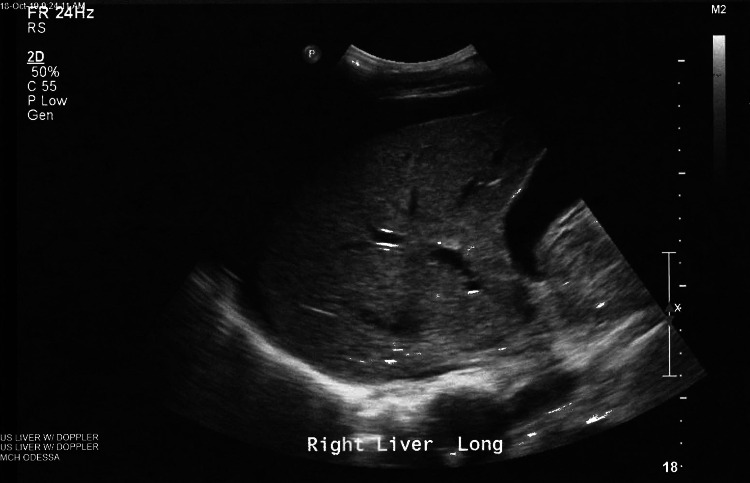
Ultrasound abdomen demonstrating normal liver echogenicity and surrounding ascitic fluid.

Ultrasound of the liver showed moderate ascites but no cirrhosis or portal vein obstruction (Figure 1). Ascitic fluid bacterial, fungal, and acid-fast bacillus (AFB) cultures were negative. Ascitic fluid analysis was consistent for exudative due to serositis from SLE. QuantiFERON-TB Gold test was negative. Laboratory workup revealed the following results (Table 1).

**Table 1:** Laboratory investigations of the patient. MCV: mean corpuscular volume; MCHC: mean corpuscular hemoglobin concentration; ESR: erythrocyte sedimentation rate; ANA: antinuclear antibody; ds-DNA: double-stranded deoxyribonucleic acid; IFA: immunofluorescence assay; anti-SSA antibody: anti-Sjögren's syndrome-related antigen A autoantibodies; hCG: human chorionic gonadotropin

Laboratory investigations	Results	Normal range or result
Leucocyte count	7200/uL	4,000-10,000/uL
Erythrocyte count	3.96 × 10^6^/uL	4.2-5.9 × 10^6^/uL
Hemoglobin	11.4 g/dL (low)	12-16 g/dL in females
Platelet count	378,000/uL	150,000-450,000/uL
MCV	83 fL	80-100 fL
MCHC	33 g/dL	32-36 g/dL
ESR	52 mm/h (high)	0-20 mm/h
ANA titers	1:80 (positive)	< 1:40 (negative)
Anti-dsDNA antibody	18 IU/mL (high)	0-9 IU/mL
ds-DNA titers by IFA	1:80 (high)	<1:10
Anti-SSA antibody	23 IU/mL (high)	0-9 IU/mL
Anti-Smith antibody	19 IU/mL (positive)	0-7 IU/mL
Complement 3 (C3)	47 mg/dL (low)	82-167 mg/dL
Complement 4 (C4)	11 mg/dL (low)	14-44 mg/dL
Hepatitis B surface antigen	Non-reactive	Non-reactive
Hepatitis B core antibody	Negative	Negative
Hepatitis C antibody	0.1 (Negative)	0-0.9 IU/mL
Human immunodeficiency virus 1/2 combo, antigen/antibody	Non-reactive	Non-reactive
Serum beta-hCG	0.2 (negative)	0-0.4 IU/L

**Figure 2:**
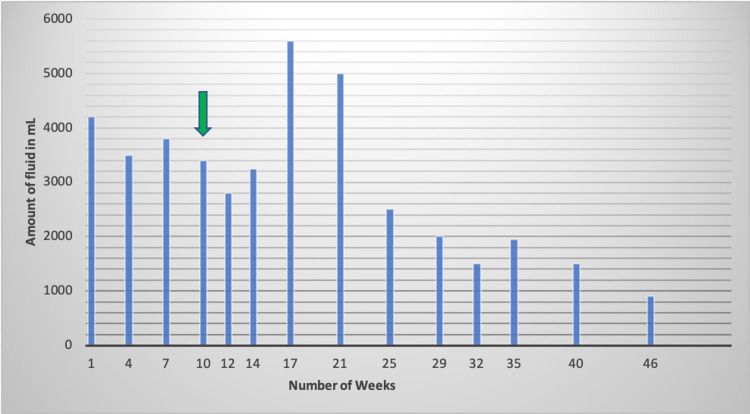
The downward pointing arrow (green) indicates the time at which the patient was started on belimumab. The x-axis shows the week number and the y-axis shows the amount of fluid drawn during the ascitic tap.

We tapered prednisone and started her on belimumab 10 mg/kg intravenous infusion every four weeks. In the following weeks, less fluid was getting drained on subsequent paracenteses as well as the frequency of paracentesis was decreased (Table 2, Figure 2). The week and the amount of fluid drained via abdominal paracentesis are listed in Table 2 and depicted in Figure 2.

**Table 2:** Amount of fluid (in mL) drawn during paracentesis after the patient was presented to the outpatient clinic. She was started on belimumab at week 10.

Week number	Amount of fluid (in mL)
Week 1	4200 mL
Week 4	3500 mL
Week 7	3800 mL
Week 10 (belimumab 10 mg/kg IV every 4 weeks approved and started)	3400 mL
Week 12	2800 mL
Week 14	3250 mL
Week 17	5600 mL
Week 21	5000 mL
Week 25	2500 mL
Week 29	2000 mL
Week 32	1500 mL
Week 35	1950 mL
Week 40	1500 mL
Week 46	900 mL

Seven months later, the titers for anti-double-stranded DNA antibodies were decreased to 1:40, C3 complement levels were low, and C4 complement levels were normal. She had four episodes of mastitis and eight episodes of urinary tract infections (UTIs) throughout the treatment. We treated her infections with appropriate antibiotics; we tapered the dose of her oral steroids ascitic fluid volume came down significantly throughout the course of belimumab therapy and the frequency of paracentesis. After one year, her abdominal pain and distension have subsided, and she no longer needed abdominal paracentesis. So, we stopped belimumab in view of infections and advised her to follow-up every three months.

## Discussion

A 25-year-old African American female with SLE was initially taking prednisone 20 mg oral daily, mycophenolate, and hydroxychloroquine with partial control of her symptoms. She presented to the medical facility multiple times with ascites that needed paracentesis every three to four weeks. We started her on intravenous belimumab every month. A few weeks later, the amount of ascitic fluid drawn during the subsequent visits was reduced, and the patient also reported significant improvement in her abdominal pain. Later, she was advised to visit the hospital for paracentesis every four to six weeks instead of three to four weeks. After seven months, the titer for anti-double-stranded DNA antibody was decreased from 1:80 to 1:40; C3 complement levels remained low but C4 levels came back normal. The patient used belimumab for one year, and her abdominal pain and swelling have subsided, and she no longer needed abdominal paracentesis. However, she had four episodes of breast infections (mastitis) and eight episodes of urinary tract infections (UTI) over the past year while using belimumab. During the same time, we tapered her prednisone from 20 mg daily to 5 mg oral daily. The first episode of UTI was reported two weeks after starting belimumab. We treated her infections with appropriate antibiotics and continued the medication as there was a significant improvement in her symptoms. Due to improvement in her abdominal symptoms, no requirement of paracentesis, and occurrence of infections, we stopped belimumab after one year and advised her to follow up in the clinic every three months. At this time we continued the rest of her medications. The learning points are as follows: (a) corticosteroids and other immunosuppressants are commonly used in the management of SLE but may not be effective in all SLE patients and cause side effects; (b) B-cells are known to play an essential role in the pathogenesis of SLE and the use of belimumab, an anti-BLys human monoclonal antibody that inhibits proliferation makes a logical choice in the management of autoantibody-positive SLE; (c) belimumab can be tried in patients presenting with SLE-associated serositis as in this patient; (d) serious and potentially fatal infections may occur during treatment with belimumab and it warrants discontinuation.

SLE is a chronic autoimmune disease that affects multiple organ systems. Most of the disease manifestations are primarily due to the formation and deposition of autoantibodies and immune complexes in body parts. Hyperactive B-lymphocytes and T-lymphocytes produce excess antibodies against the antigens present on the surface of apoptotic cells [6]. B-lymphocytes are stimulated by apoptotic cellular material to produce autoantibodies, resulting in immune complexes formation and deposition. These immune complexes are deposited in the tissues and induce an inflammatory reaction that ultimately results in tissue damage [6]. Most of the clinical manifestations of the disease are due to this abnormal inflammatory response. As B-cells play a significant role in the disease process, monoclonal antibodies such as rituximab and belimumab targeting B-cells are being used in the treatment.

Belimumab is a fully humanized IgG1-λ that blocks the binding of soluble B-lymphocyte stimulator protein (BLyS) to its receptor on B-lymphocytes and thus its activity [8,9]. BLyS is a growth factor that is needed for B-lymphocyte maturation, survival, and activation [10]. It is also required for the maturation of B-cells into plasma cells and immunoglobulin production [10]. At least half of the patients with SLE are observed to have elevated plasma levels of soluble BlyS, and belimumab was observed to improve both serological and clinical findings in SLE patients [11,12]. Thus, belimumab is widely being used in the management of SLE. However, the spectrum presentations that benefit belimumab need to be elucidated. The adverse effects of belimumab include hypersensitivity or infusion reactions, infections, psychiatric disturbances like depression, suicidal behavior, and increased risk of malignancy and progressive multifocal leukoencephalopathy. Currently, belimumab is approved to manage autoantibody-positive SLE (antinuclear antibodies and anti-DNA antibodies) with a high degree of disease activity despite receiving standard therapy [13]. It is also used for the management of mucocutaneous and musculoskeletal manifestations and may be used in any patient without severe lupus nephritis or neuropsychiatric manifestations [14].

## Conclusions

There is not enough evidence to suggest intravenous belimumab as an effective therapy for chronic serositis associated with systemic lupus erythematosus in our literature search. Our patient is a 25-year-old African American female with a positive antinuclear antibody (ANA), double-stranded DNA antibody (dsDNA Ab), Smith antibody (Smith Ab), and Sjogren's syndrome A (SSA) antibody. She is on lifelong anticoagulation therapy with coumadin due to antiphospholipid antibody syndrome. She also has a history of lupus nephritis that remained stable over many years.

She developed chronic serositis that required frequent paracentesis. After starting belimumab, her serositis improved, we managed to taper her steroids from 20 mg daily to 5 mg daily. She developed frequent infections during the same time, mastitis, and frequent UTIs. We will not know if her frequent infections are due to prednisone, mycophenolate, belimumab, or because of the combination of her immunosuppressive medications. We noticed an improvement in serositis in terms of symptoms, amount of ascitic fluid, and frequency of paracentesis. Based on our case report, it looks like belimumab has a role in treating chronic serositis associated with systemic lupus patients. But clinicians should be vigilant and watch for frequent infections while on belimumab therapy.
